# Differentiating Spotted Fever From Scrub Typhus Based on Clinical and Laboratory Features: A Retrospective Cohort Study From South India

**DOI:** 10.1093/ofid/ofag264

**Published:** 2026-05-01

**Authors:** Nitin Gupta, Tirlangi Praveen Kumar, Kavita Salian, Prithvishree Ravindra, Rachana Bhat, Steven Van Den Broucke, Erika Vlieghe, Emmanuel Bottieau

**Affiliations:** Department of Infectious Diseases, Kasturba Medical College, Manipal Academy of Higher Education, Manipal, India; Department of Clinical Sciences, Institute of Tropical Medicine, Antwerp, Belgium; University of Antwerp, Antwerp, Belgium; Department of Infectious Diseases, Kasturba Medical College, Manipal Academy of Higher Education, Manipal, India; Department of Infectious Diseases, Kasturba Medical College, Manipal Academy of Higher Education, Manipal, India; Department of Emergency Medicine, Kasturba Medical College, Manipal Academy of Higher Education, Manipal, India; Department of Emergency Medicine, Kasturba Medical College, Manipal Academy of Higher Education, Manipal, India; Department of Clinical Sciences, Institute of Tropical Medicine, Antwerp, Belgium; Department of Clinical Sciences, Institute of Tropical Medicine, Antwerp, Belgium; University of Antwerp, Antwerp, Belgium; University Hospital Antwerp, Antwerp, Belgium; Department of Clinical Sciences, Institute of Tropical Medicine, Antwerp, Belgium

**Keywords:** acute undifferentiated febrile illness, India, rickettsial infections, scrub typhus, spotted fever

## Abstract

**Background:**

Scrub typhus and spotted fever are the two most common rickettsial infections in India. Both conditions differ, however, in terms of epidemiological risk, clinical presentation, and prognosis. We aimed to compare the clinical and laboratory characteristics of patients diagnosed with scrub typhus or spotted fever to identify disease indicators that are useful in low-resource endemic settings.

**Methods:**

We conducted a retrospective cohort study among adults admitted with undifferentiated fever lasting 5–15 days at a tertiary care hospital in coastal Karnataka, India, between January 2017 and December 2023. Probable spotted fever and probable scrub typhus were diagnosed by single positive serology, using the Weil–Felix test and IgM ELISA, respectively. Patients with coinfection between the two study conditions or with any other confirmed pathogen were excluded from the analysis. Binary logistic regression was performed with spotted fever as the dependent variable and scrub typhus as the referent category.

**Results:**

Of 1045 patients with undifferentiated fever, 94 met the study criteria for probable spotted fever and 643 for probable scrub typhus. Probable spotted fever was observed throughout the year, whereas probable scrub typhus showed a marked monsoon and post-monsoon predominance, from June to November. Rash (39.4% vs 11.0%, *P* < .001) and leucocytosis (46.8% vs 26.6%, *P* < .001) were significantly more frequent in probable spotted fever than in probable scrub typhus. In contrast, splenomegaly (21.3% vs 37.5%, *P* = .002), lymphadenopathy (2.1% vs 11.8%, *P* = .004), lung involvement (34.0% vs 62.2%, *P* < .001), and hypoalbuminemia (55.6% vs 75.7%, *P* < .001) were less frequent in probable spotted fever than in probable scrub typhus. In multivariable analysis with spotted fever as the outcome, rash (adjusted odds ratio [aOR], 5.69, *P* < .001) and leucocytosis (aOR, 3.67, *P* < .001) were independently associated with higher odds of probable spotted fever. In contrast, monsoon or post-monsoon presentation (aOR, 0.48, *P* = .006), splenomegaly (aOR, 0.45, *P* = .008), lymphadenopathy (aOR, 0.09, *P* = .002), lung involvement (aOR, 0.29, *P* < .001), and hypoalbuminemia (aOR, 0.45, *P* = .003) were associated with lower odds of probable spotted fever relative to probable scrub typhus. There was no significant difference in day-7 mortality between the two groups.

**Conclusions:**

Probable scrub typhus and probable spotted fever can be differentiated by a set of epidemiological, clinical, and first-line laboratory variables. These findings should improve the bedside diagnostic approach while awaiting further multicenter prospective validation.

## Teaser

In a large cohort of patients with rickettsial infections in South India, simple clinical and laboratory features helped distinguish scrub typhus from spotted fever. Rash and leucocytosis favored spotted fever, whereas seasonal presentation, splenomegaly, lymphadenopathy, lung involvement, and hypoalbuminemia pointed toward scrub typhus.

## Graphical Abstract

**Graphical Abstract:**
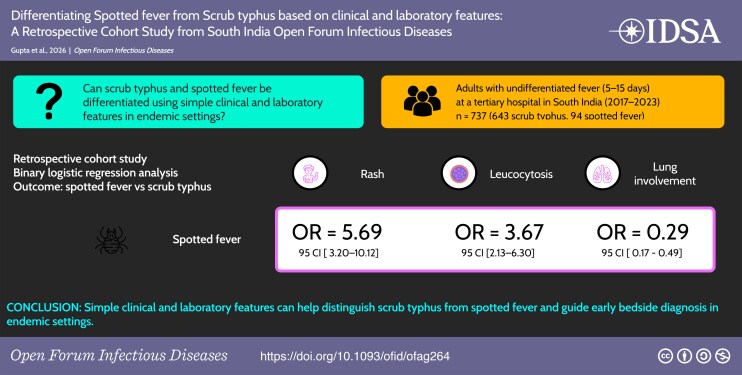
This graphical abstractis also available at Tidbit: https://tidbitapp.io/tidbits/differentiating-spotted-fever-from-scrub-typhus-based-on-clinical-and-laboratory-features-a-retrospective-cohort-study-from-south-india-open-forum-infectious-diseases

Rickettsial infections are increasingly recognized as significant causes of undifferentiated febrile (UF) illnesses in India [1, 2]. In this context, two closely related genera within the family *Rickettsiaceae* account for the majority of cases: *Orientia tsutsugamushi*, the agent of scrub typhus, and members of the Spotted Fever Group (SFG), most commonly *Rickettsia conorii* and *Candidatus Rickettsia kellyi*, which are responsible for spotted fever in India [1, 3, 4]. Other spotted fever group rickettsiae, such as *Rickettsia rickettsii*, *Rickettsia africae*, *Rickettsia australis*, *Rickettsia parkeri*, and *Rickettsia akari*, as well as typhus group rickettsiae, namely *Rickettsia typhi* and *Rickettsia prowazekii*, have not been documented or have only been sporadically reported in India and are not recognized as established endemic causes of acute febrile illness in this region [5]. Similarly, data on *Coxiella burnetii* infection remain limited [6]. Although historically grouped with rickettsial organisms due to its intracellular nature, *C. burnetii* is taxonomically distinct from the genus *Rickettsia*. Sporadic cases of Q fever have been reported, typically associated with livestock or farm exposure, and it is not commonly identified as a major cause of febrile illness in southern India [6].

Within this epidemiologic landscape, scrub typhus and spotted fever represent the two principal rickettsial syndromes encountered in clinical practice in southern India. Both conditions typically present with nonlocalizing manifestations such as fever, headache, and myalgia, and may progress to multiorgan involvement if not promptly recognized and treated with doxycycline or azithromycin [5]. Despite this clinical overlap, important differences exist in their pathophysiology, vector ecology, organ tropism, and patterns of complications. Scrub typhus is transmitted by larval trombiculid mites (chiggers), whereas hard ticks transmit spotted fever [7, 8]. In South India, both ecological niches are relevant, and seasonal rainfall, vegetation, agricultural exposure, and peridomestic animal contact may plausibly influence the pattern of human exposure [7, 8]. Scrub typhus is among the most frequently reported causes of febrile illness in southern India and in the Himalayan and sub-Himalayan regions [9]. A systematic review found that 25.3% of all acute undifferentiated febrile illnesses in India were caused by scrub typhus. In contrast, data on spotted fever from India remain limited, largely confined to small regional series or case reports [2, 5, 10, 11], contributing to its under-recognition, especially in rural settings with limited diagnostic access. More broadly, rickettsial infections remain neglected despite their substantial burden in tropical regions, reflecting gaps in surveillance, laboratory capacity, and research prioritization [12]. Owing to differences in epidemiology and potential variations in disease progression and prognosis, timely differentiation between scrub typhus and spotted fever is clinically significant. Accordingly, this study compared the clinical and laboratory characteristics of hospitalized adults with scrub typhus or spotted fever in coastal South India to identify independent factors associated with each condition.

## METHODS

### Study Design and Setting

This study was conducted at a tertiary care teaching and referral hospital in coastal Karnataka, India, serving both residents and referred patients from neighboring districts. Adults presenting with prolonged undifferentiated fever are typically evaluated through routine first-line investigations for common endemic infections, including malaria, dengue, leptospirosis, and bacteremia; rickettsial testing is usually pursued when these are negative or when clinical suspicion persists. Because molecular assays were unavailable in routine care during the study period, serology frequently informed bedside diagnostic categorization. The study was approved by the Institutional Ethics Committee (IEC number: IEC1 12/2025). The requirement for informed consent was waived owing to the retrospective design and use of anonymized data.

### Patient Screening and Eligibility

All adults aged 15 years or above who were admitted with undifferentiated fever between January 2017 and December 2023 were screened. For this study, undifferentiated fever was defined as a reported or documented fever of 5–15 days’ duration without a clear localizing source, as determined by history taking and clinical examination. This time frame was chosen because the diagnosis could only be made by serology. The diagnosis of spotted fever and scrub typhus was made in accordance with the definitions of *probable disease* specified in the national guidelines for rickettsial infections in India [13]. These case definitions were applied despite their recognized limitations, as molecular assays for both pathogens were unavailable in the study setting, and follow-up samples for paired serology were obtained infrequently. This approach reflects real-world diagnostic constraints in endemic, resource-limited settings, where single-sample serology remains the primary basis for clinical decision-making, and is consistent with national guidelines that recommend these tests in the absence of molecular assays. Additionally, only patients with negative blood cultures at admission and negative microbiological tests for dengue (NS1 antigen and/or IgM ELISA), malaria (quantitative buffy coat), and leptospirosis (IgM ELISA) were retained for this analysis. Those patients in whom any other microbiological diagnosis was confirmed, as well as those in whom both spotted fever and scrub typhus serologies were positive, were also excluded. Those patients with incomplete clinical or laboratory data were excluded.

### Diagnostic Definitions

Probable spotted fever was diagnosed using the Weil–Felix test, defined as OX2 or OX19 agglutination titers ≥1:160 (Tulip Diagnostics, India); at such thresholds, the test is recognized to have low sensitivity but relatively high specificity, although performance varies considerably across rickettsial groups and settings [14]. Laboratory diagnosis of probable scrub typhus was based on a positive IgM ELISA for *Orientia tsutsugamushi* (Scrub Typhus Detect™, InBios International Inc., USA), which in Indian settings has demonstrated a sensitivity of 97% (95% CI, 83%–100%) and specificity of 100% (95% CI, 88%–100%) when evaluated against composite reference standards, as reported previously by our group [15]. In a previous randomized diagnostic study from our center, the median turnaround time for scrub typhus IgM ELISA was 50 hours (IQR, 30–72 hours), underscoring the delay inherent in serology-based diagnosis in routine practice [16].

### Sample Size Calculation

Based on the primary objective of developing a multivariable model identifying factors independently associated with spotted fever among patients with rickettsial infections, sample size considerations were guided by an events-per-variable framework as described by Riley et al [17]. In addition to estimating the required number of outcome events, we prespecified the maximum number of candidate parameters to retain in the final model to limit overfitting. Assuming that no more than seven variables would be eligible for retention, we targeted a minimum of 15 outcome events per variable to ensure adequate model stability, reduce optimism in effect estimates, and maintain reliable regression coefficients [17]. In a study conducted in the same region of India, among 48 patients who tested positive for either scrub typhus or spotted fever (but not both), 9 (19%) were diagnosed with spotted fever [18]. Using this prevalence and accounting for an expected 15% of missing or incomplete data, the required total sample size was estimated as 737 participants.

### Outcomes

Clinical outcomes were assessed during the index hospitalization using data extracted from the medical records. The primary outcome for the regression analysis was a diagnosis of probable spotted fever, with probable scrub typhus serving as the referent category. In addition, we evaluated disease severity relevant to patient management. A formal composite severity definition was not prespecified in this retrospective analysis. To avoid post hoc misclassification, we report objective markers of severe disease and organ support, including shock, mechanical ventilation, dialysis, and day-7 mortality.

### Data Collection

Data were extracted from hospital records using a structured data collection tool. Demographic details, including age, sex, and occupation, were recorded. Patients were categorized by seasonality, with those presenting between June and November considered monsoon/post-monsoon presentations. Clinical features and features suggestive of organ involvement were recorded. Lung involvement was defined as the presence of radiographic infiltrates on chest X-ray or the requirement for supplemental oxygen. Central nervous system involvement was defined by altered sensorium, meningeal signs, seizures, focal neurological deficits, cerebrospinal fluid pleocytosis, or leptomeningeal enhancement on neuroimaging. Heart involvement was defined as myocarditis, characterized by elevated troponin levels or new echocardiographic changes, and kidney involvement (acute kidney injury) was defined according to the KDIGO (Kidney Disease: Improving Global Outcomes) criteria. Leucocytosis was defined as a total leukocyte count exceeding 11 000 cells/µL, while thrombocytopenia was defined as a platelet count below 150 000/µL. Transaminitis (elevated liver enzymes) was defined as an elevation of either aspartate aminotransferase or alanine ALT above 40 U/L. Hypoalbuminemia was defined as a serum albumin concentration below 3.5 g/dL, and elevated C-reactive protein (CRP) was defined as a level above 6 mg/L.

### Data Analysis

Data were analyzed using IBM SPSS Statistics for Windows, Version 26.0 (IBM Corp., Armonk, NY, USA). Continuous variables were assessed for normality using visual inspection of histograms and the Shapiro–Wilk test. Normally distributed variables were summarized as mean ± standard deviation and compared using Student's t-test. In contrast, skewed variables were summarized as medians with interquartile ranges (IQRs) and compared using the Mann–Whitney *U* test. Categorical variables were expressed as frequencies and percentages and compared using the *χ*² or Fisher's exact test, as appropriate. To identify factors independently associated with spotted fever, a binary logistic regression analysis was performed with spotted fever as the dependent variable. Statistically significant variables (*P* value < .05) from the univariate analysis were entered into the multivariable model. Multicollinearity was assessed using correlation matrices and variance inflation factors, and highly correlated variables were excluded to ensure model stability. Model refinement was conducted using a backward stepwise elimination procedure, sequentially removing the least significant variables. A two-sided *P*-value threshold of .05 was used to retain models, preserving parsimony and minimizing the risk of overfitting. Model calibration, defined as the agreement between observed and expected outcomes, was assessed using the Hosmer–Lemeshow goodness-of-fit test. Model discrimination was evaluated using the area under the receiver operating characteristic (ROC) curve (AUC), also known as the C-statistic. Explained variance was estimated using the Nagelkerke *R*^2^. Model fit and parsimony were further assessed using the Akaike Information Criterion (AIC) and Bayesian Information Criterion (BIC). Odds ratios (ORs) with 95% CIs were calculated to quantify the strength of associations.

## RESULTS

**Figure 1.**
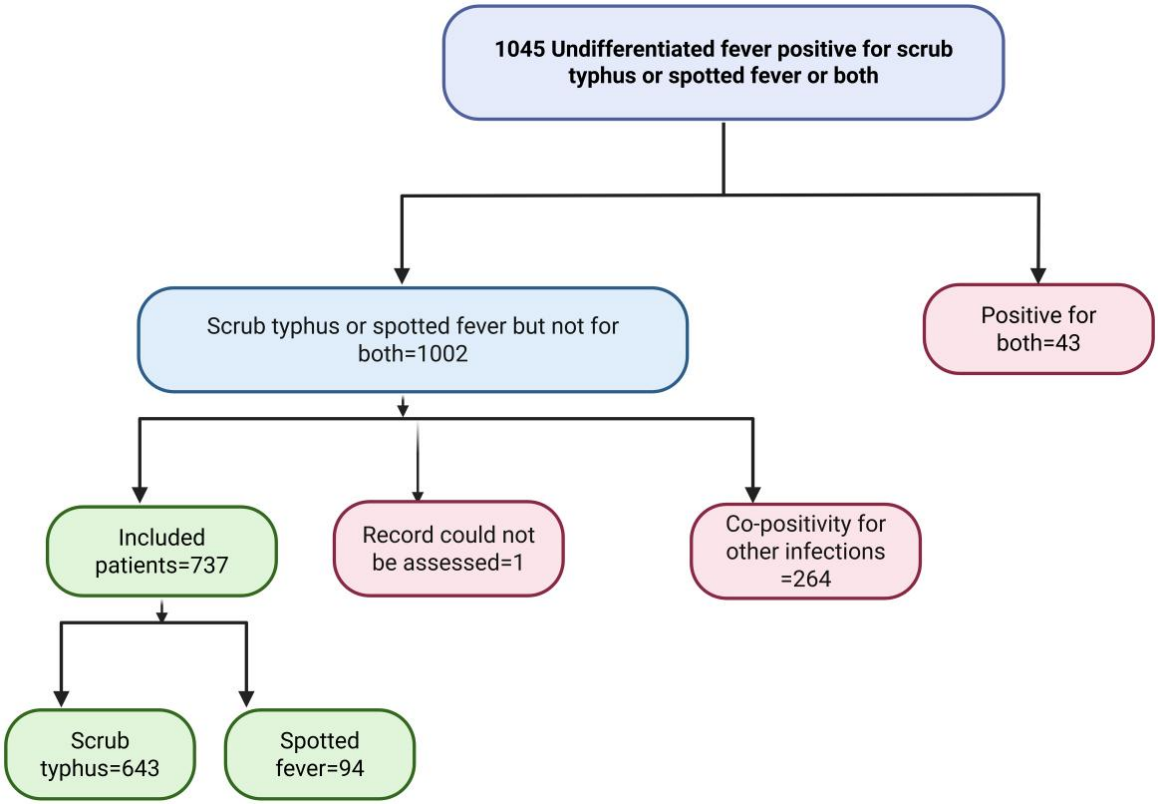
Flow chart illustrating inclusion and exclusion of patients with undifferentiated fever (duration 5–15 d) who tested serologically positive for scrub typhus, spotted fever, or both. Abbreviations: KFD, Kyasanur Forest Disease.

A total of 1045 UF patients (with a fever duration of 5–15 days) tested serologically positive for either scrub typhus, spotted fever, or both (Figure 1). Of these, 43 patients were excluded as they were positive for both infections. An additional 264 patients were excluded due to concomitant positivity for other infections (leptospirosis, n = 201; dengue, n = 27; Kyasanur Forest Disease, n = 2; malaria, n = 1; Other infections, n = 33). One patient was excluded because the original medical record could not be retrieved. The final analysis compared 643 patients with probable scrub typhus and 94 with probable spotted fever.

Probable spotted fever occurred throughout the year, whereas probable scrub typhus showed a marked seasonal trend, with the majority of cases occurring during the monsoon and post-monsoon months, June to November. Patients with spotted fever were younger than those with probable scrub typhus, with a median age of 39.5 years (IQR, 29.0 to 52.0) versus 46.0 years (IQR, 35.0 to 57.0; *P* = .003). The duration of illness at presentation was similar, with a median of 7.0 days (IQR, 6.0 to 10.0) in spotted fever and 7.0 days (IQR, 5.0 to 10.0) in probable scrub typhus (*P* = .218). There was no significant difference between the two infections in gender distribution (60.6% vs 58.2%; *P* = .649), outdoor occupation (67.5% vs 64.9%; *P* = .646), or diabetes mellitus (26.6% vs 25.5%; *P* = .828) (Table 1). Rash (39.4% vs 11.0%; *P* < .001) and leucocytosis (46.8% vs 26.6%; *P* < .001) were significantly more frequent in probable spotted fever. In contrast, icterus (17.0% vs 28.2%; *P* = .023), splenomegaly (21.3% vs 37.5%; *P* = .002), lymphadenopathy (2.1% vs 11.8%; *P* = .004), lung involvement (34.0% vs 62.2%; *P* < .001), thrombocytopenia (50.0% vs 67.6%; *P* = .001), transaminitis (78.7% vs 90.3%; *P* < .001), and hypoalbuminemia (55.6% vs 75.7%; *P* < .001) were less common in probable spotted fever. Eschar was not observed in patients with probable spotted fever but was present in 15.2% of those with probable scrub typhus (*P* < .001). Shock, kidney involvement, and heart involvement occurred at similar frequencies in the two groups. Elevated CRP levels were highly prevalent in both probable spotted fever and probable scrub typhus (94.1% vs 96.6%; *P* = .391) (Table 1).

**Table 1.** Comparison of Demographic, Clinical, and Laboratory Features and Outcomes Between Spotted Fever and Scrub Typhus

Section	Variable	Probable Spotted Fever (n = 94)	Probable Scrub Typhus (n = 643)	*P V*alue
Demography	Age, years, median (IQR)	39.5 (29.0–52.0)	46.0 (35.0–57.0)	.003*
	Monsoon and post-monsoon season, June–November	46 (48.9%)	476 (74.0%)	<.001*
	Male gender	57 (60.6%)	374 (58.2%)	.649
	Outdoor occupation	56/83 (67.5%)	340/524 (64.9%)	.646
	Diabetes mellitus	25 (26.6%)	164 (25.5%)	.828
Clinical profile	Duration of illness, days, median (IQR)	7.0 (6.0–10.0)	7.0 (5.0–10.0)	.218
	Headache	40 (42.6%)	230 (35.8%)	.202
	Rash	37 (39.4%)	71 (11.0%)	<.001*
	Bleeding manifestations	6 (6.4%)	23 (3.6%)	.191
	Icterus	16 (17.0%)	181 (28.2%)	.023*
	Splenomegaly	20 (21.3%)	241 (37.5%)	.002*
	Lymphadenopathy	2 (2.1%)	76 (11.8%)	.004*
	Eschar	0 (0%)	98 (15.2%)	<.001*
	Shock	6 (6.4%)	69 (10.7%)	.193
	CNS involvement	21 (22.3%)	95 (14.8%)	.060
	Lung involvement	32 (34.0%)	400 (62.2%)	<.001*
	Kidney involvement	27 (28.7%)	179 (27.8%)	.858
	Heart involvement	17 (18.1%)	120 (18.7%)	.893
Laboratory profile	Leucocytosis	44/94 (46.8%)	171/643 (26.6%)	<.001*
	Thrombocytopenia	47/94 (50.0%)	404/598 (67.6%)	.001*
	Transaminitis	74/94 (78.7%)	577/639 (90.3%)	<.001*
	Hypoalbuminemia	50/90 (55.6%)	461/609 (75.7%)	<.001*
	Elevated CRP	48/51 (94.1%)	337/349 (96.6%)	.391
Outcomes	Mechanical ventilation	9 (9.6%)	102 (15.9%)	.111
	Dialysis	2 (2.1%)	18 (2.8%)	.708
	Day-7 mortality	0 (0%)	12 (1.9%)	.182

Data are presented as numerator/denominator (percentage), unless otherwise indicated. Age and duration of illness are presented as median (interquartile range). The monsoon and post-monsoon period were defined as June to November based on regional climatology in the Udupi district. Outdoor occupations included farming, fishing, or other field-based work. Shock was defined as systolic blood pressure <90 mmHg or need for vasopressor support. Lung involvement was defined by chest X-ray infiltrates or need for supplemental oxygen, and CNS involvement by altered sensorium, seizures, meningeal signs, or cerebrospinal fluid pleocytosis. Kidney involvement indicated acute kidney injury as per KDIGO criteria. Transaminitis was defined as ALT or AST >40 U/L, hypoalbuminemia as serum albumin <3.5 g/dL, leucocytosis as total leukocyte count >11 000/µL, thrombocytopenia as platelet count <150 000/µL, and elevated C-reactive protein (CRP) as >6 mg/L. Mechanical ventilation, dialysis, and day 7 mortality were recorded as short-term outcome measures. *P* values were calculated using the *χ*² test or Fisher's exact test for categorical variables and the Mann–Whitney *U* test for continuous variables, with *P* < .05 considered statistically significant. Abbreviations: ALT, alanine aminotransferase; AST, aspartate aminotransferase; CNS, central nervous system; CRP, C-reactive protein; IQR, interquartile range; KDIGO, Kidney Disease, Improving Global Outcomes.

An asterisk (*) denotes variables with *P* < .05 on univariate comparison and therefore considered eligible for multivariable assessment, except eschar, which showed complete separation and was not entered into the final multivariable model.

On multivariable logistic regression, rash (aOR, 5.69; 95% CI, 3.20–10.12; *P* < .001) and leucocytosis (aOR, 3.67; 95% CI, 2.13–6.30; *P* < .001) were independently associated with higher odds of probable spotted fever. In contrast, monsoon/post-monsoon presentation (aOR, 0.48; 95% CI, 0.29–0.81; *P* = .006), splenomegaly (aOR, 0.45; 95% CI, 0.25–0.81; *P* = .008), lymphadenopathy (aOR, 0.09; 95% CI, 0.02–0.41; *P* = .002), lung involvement (aOR, 0.29; 95% CI, 0.17–0.49; *P* < .001), and hypoalbuminemia (aOR, 0.45; 95% CI, 0.26–0.77; *P* = .003) were associated with lower odds of probable spotted fever (Table 2). The final multivariable model demonstrated acceptable calibration, as indicated by a nonsignificant Hosmer–Lemeshow goodness-of-fit test (χ^2^ = 7.10, *P* = .526). The model showed good discriminatory performance (AUC, 0.837; 95% CI, 0.794–0.880) and explained a moderate proportion of variance (Nagelkerke *R*^2^ = 0.317). Model fit and parsimony were further assessed using the AIC (422.7) and BIC (459.1).

**Table 2.** Logistic Regression Analysis for Factors Associated With Spotted Fever Among Patients With Rickettsial Infections

Variable	Unadjusted OR (95% CI)	Adjusted OR	95% CI For Adjusted OR	*P V*alue	Status In Final Model
Monsoon and post-monsoon season, June–November	0.336 (0.216–0.523)	0.483	0.288–0.808	.006	Retained
Rash	5.230 (3.230–8.460)	5.689	3.198–10.123	<.001	Retained
Icterus	0.524 (0.298–0.921)	—	—	—	Removed during backward elimination
Splenomegaly	0.451 (0.268–0.758)	0.445	0.245–0.808	.008	Retained
Lymphadenopathy	0.162 (0.039–0.672)	0.091	0.020–0.411	.002	Retained
Eschar	0.029 (0.002–0.476)	—	—	—	Not entered due to complete separation
Lung involvement	0.314 (0.199–0.494)	0.286	0.166–0.492	<.001	Retained
Leucocytosis	2.429 (1.562–3.776)	3.665	2.133–6.295	<.001	Retained
Thrombocytopenia	0.480 (0.310–0.745)	—	—	—	Removed during backward elimination
Transaminitis	0.398 (0.227–0.695)	—	—	—	Removed during backward elimination
Hypoalbuminemia	0.401 (0.255–0.633)	0.445	0.259–0.766	.003	Retained

Odds ratios are shown with spotted fever as the outcome of interest and scrub typhus as the referent category. Variables with *P* < .05 in the univariate analysis were considered for inclusion in the multivariable model. Eschar was not included in the multivariable model because no patients with spotted fever had an eschar, leading to complete separation. Abbreviations: OR, odds ratio; CI, confidence interval.

Model performance metrics: Nagelkerke *R*^2^ = 0.317; Hosmer–Lemeshow *P* = .526; AUC = 0.837 (95% CI 0.794–0.880); AIC = 422.7; BIC = 459.1.

All patients received anti-rickettsial therapy with doxycycline, azithromycin, or a combination of both. Outcomes related to organ support and early mortality were comparable between probable spotted fever and probable scrub typhus (Table 1). The need for mechanical ventilation did not differ significantly between patients with probable spotted fever (9.6%) and those with probable scrub typhus (15.9%; *P* = .111). Similarly, dialysis was infrequent in both groups, being required in 2.1% of probable spotted fever cases and 2.8% of probable scrub typhus cases (*P* = .708). Day-7 mortality was low overall: no patient with probable spotted fever died, whereas 12 patients with probable scrub typhus (1.9%) died, although this difference was not statistically significant (*P* = .182).

## DISCUSSION

In this retrospective cohort study of serologically defined probable rickettsial disease among patients with undifferentiated fever in coastal Karnataka, India, probable spotted fever was observed year-round. In contrast, probable scrub typhus was the predominant infection and showed a marked seasonal trend, with a peak in the post-monsoon season. In multivariable analysis, rash and leucocytosis were independently associated with higher odds of probable spotted fever. In contrast, monsoon/postmonsoon presentation, splenomegaly, lymphadenopathy, lung involvement, and hypoalbuminemia were associated with lower odds of probable spotted fever relative to probable scrub typhus. These readily identifiable clinical and laboratory features may help distinguish the two major rickettsial syndromes in endemic settings. However, the findings should be interpreted cautiously, given the limitations of serology-based diagnosis.

Although both probable scrub typhus and probable spotted fever respond to doxycycline or azithromycin, distinguishing between them remains clinically and epidemiologically important [19, 20]. The two infections differ in vector ecology, seasonality, environmental exposure, and geographic distribution, with scrub typhus transmitted by *Leptotrombidium* mites and spotted fever by hard ticks [5]. These differences have direct implications for surveillance and prevention, as identifying the predominant rickettsial syndrome in a given setting can improve understanding of local transmission dynamics and guide vector control measures. For example, recognition of probable spotted fever in areas previously considered scrub typhus–dominant may prompt targeted tick surveillance and environmental interventions. The phenotypic differences observed in our cohort may also inform bedside assessment while confirmatory diagnostics remain unavailable; greater pulmonary involvement may raise suspicion of probable scrub typhus and warrant closer respiratory monitoring, whereas rash and tick-associated exposure may increase suspicion of probable spotted fever. Better characterization of spotted fever is also important for improving recognition of a likely underdiagnosed infection in India and for advocating greater diagnostic availability, as its true burden is probably obscured by limited awareness and underreporting. More broadly, in resource-limited settings, the practical diagnostic challenge extends beyond distinguishing probable scrub typhus from probable spotted fever alone, because both syndromes overlap clinically with dengue, chikungunya, leptospirosis, malaria, enteric fever, acute Q fever, and other zoonotic or arboviral illnesses. Distinguishing rickettsial disease from these alternative diagnoses has important implications for etiological classification, timely initiation of doxycycline or azithromycin, anticipation of complications, and prioritization of supportive care when access to definitive diagnostic tools is limited [12].

The distinct temporal distribution observed in this study likely reflects differences in the ecology and activity of their vectors. Scrub typhus, transmitted by *Leptotrombidium* mites, peaks during humid monsoon months when mite populations proliferate in dense vegetation [21]. In contrast, spotted fever, transmitted by hard ticks, is more evenly distributed throughout the year and may exhibit increased incidence during cooler, drier months, when outdoor human activity facilitates tick exposure [5, 22]. Awareness of these patterns helps clinicians integrate seasonality as a useful diagnostic cue in patients presenting with undifferentiated fever. Clinically, the predominance of rash in probable spotted fever contrasts with the hepatic, pulmonary, and reticuloendothelial involvement in probable scrub typhus [23, 24]. Although eschar was specific to probable scrub typhus, it did not retain statistical significance in multivariate analysis, likely due to its low prevalence and its strong association with probable scrub typhus, which contributed more to model classification through other correlated variables [25]. The observed differences in clinical presentation may reflect underlying pathophysiological distinctions between *Rickettsia* spp. and *Orientia tsutsugamushi* at the level of the vascular endothelium [26]. Spotted fever group rickettsiae exhibit pronounced endothelial tropism, invading and proliferating within vascular endothelial cells, leading to widespread microvascular inflammation, increased vascular permeability, and direct endothelial injury [23]. In contrast, although *O. tsutsugamushi* also infects endothelial cells, it exhibits a broader tropism, including monocytes, macrophages, and the reticuloendothelial system, with prominent pulmonary and systemic inflammatory involvement [27].

Several important limitations should be considered when interpreting our findings. First, the retrospective design carries an inherent risk of incomplete documentation and missing data, particularly for clinical signs such as rash and eschar that depend on careful examination and explicit recording. The extent of under-documentation cannot be precisely quantified and may have influenced the reported frequencies of certain clinical features. Second, this was a single-center study conducted in a tertiary-care hospital. Although such settings are appropriate for evaluating prolonged undifferentiated febrile illness, monocentric recruitment may limit the generalizability of our findings to other healthcare contexts. In particular, this may introduce spectrum bias, in which the distribution and severity of disease in a tertiary-care population differ from those in primary- or secondary-care settings, potentially influencing the observed clinical associations. Third, diagnoses were based on single-sample serology, which carries inherent limitations. Serologic cross-reactivity within the Rickettsiaceae family and background seropositivity in endemic regions may result in some degree of misclassification, particularly in the absence of molecular confirmation. Although *Coxiella burnetii* is taxonomically distinct from *Rickettsia*, it was not routinely tested in this cohort; therefore, undetected Q fever cannot be completely excluded, although it is not recognized as a common cause of UF in our setting. In addition, single-sample serology does not permit the determination of infection timing or pathogen burden and cannot reliably distinguish acute from recent exposure. Some competing diagnoses within the tropical UF spectrum, particularly leptospirosis and other infections diagnosed using single-sample serology or IgM-based assays, may themselves have imperfect specificity. This creates a bidirectional risk of misclassification and may have led to underestimation of both probable spotted fever and probable scrub typhus in routine practice. These factors constrain etiological certainty at the individual patient level but do not invalidate the observed clinical and epidemiologic patterns within the defined diagnostic categories, which reflect real-world practice in resource-limited endemic settings. Finally, the comparative analysis between probable scrub typhus and probable spotted fever represents a structured epidemiologic contrast rather than a comprehensive clinical decision framework. In routine clinical practice, the differential diagnosis of UF extends beyond rickettsioses alone. Accordingly, the identified associations should be interpreted as context-specific indicators within serologically defined groups and require prospective, multicenter validation incorporating improved diagnostic confirmation before broader clinical application.

Despite these limitations, the study also has notable strengths. It includes a relatively large cohort of rickettsial infections from a region where these diseases are endemic and under-recognized, providing valuable epidemiological and clinical insights. Standardized clinical definitions and a uniform data extraction template were used to reduce heterogeneity in recorded variables. Multivariable logistic regression, implemented with prespecified limits on model complexity based on an events-per-variable framework and accompanied by formal assessment of calibration, discrimination, and information criteria, allowed identification of independent associations while minimizing overfitting and model optimism. Furthermore, although serology is imperfect, we attempted to mitigate diagnostic misclassification by applying stringent eligibility criteria and excluding samples with overlapping positive results for other common causes of acute febrile illness. Taken together, these efforts enhance the robustness of the findings and provide pragmatic, bedside-relevant cues for clinicians, while acknowledging that further prospective, multi-center studies with improved diagnostic tools are needed to refine these observations and improve generalizability.

In summary, probable scrub typhus and probable spotted fever, though clinically overlapping, have some discriminative epidemiological, clinical, and laboratory features that can be useful in the diagnostic approach at the bedside. Prospective multicenter validation incorporating diagnostics with direct pathogen demonstration and standardized clinical documentation is warranted to refine and integrate the identified set of discriminative findings into a model for broader application in endemic regions.
